# Anti-idiotype antibodies in cancer treatment

**DOI:** 10.3389/fonc.2013.00037

**Published:** 2013-02-26

**Authors:** Daniel E. Gomez, Ana M. Vázquez, Daniel F. Alonso, Amparo Macías

**Affiliations:** ^1^Department of Science and Technology, Universidad Nacional de QuilmesBuenos Aires, Argentina; ^2^Centro de Inmunologia MolecularLa Havana, Cuba

Anti-idiotype antibodies (anti-Id Abs) are antibodies to idiotopes that are located in the variable region, including the antigen binding site, of another antibody. When the last is the case, these anti-Id Abs can act as surrogates of the original antigen. The capability of anti-Id Abs to modulate the immune response has been the basis for the development of anti-Id vaccines against different antigens, including tumor-associated antigens. Over the years, its use in cancer has been demonstrated as effective and promising. This book “Anti-idiotype antibodies in cancer treatment” resumes the latest findings in the field. The book starts with an opinion article by Gomez et al. (2012), whereas the authors discuss a method for prioritization of cancer antigens that paves the way to take more rational, informed decisions in vaccine development. Following, we will find a number of reviews that conform a complete updating on the subject. The first one by Kieber-Emmons et al. (2012) explore the concept of anti-Id Abs with its achievements and drawbacks. Following, Ladjemi (2012) focuses on recent achievements of use of anti-Id Abs as cancer vaccines in solid tumors. López-Requena et al. (2012) focus on the role of anti-Id vaccination in cancer management and on the current developments used to foster anti-idiotypic B and T cell responses. Vázquez et al. (2012a,b) deeply analyze the immunological mechanisms involved in the use of these antibodies, while Vázquez et al. (2012a,b) focus on racotumomab, an anti-Id vaccine already in Phase III clinical trials. Finally, Fredriksen et al. (2012) present a hypothetical model for how the APC-targeted vaccine molecules enhance Id-specific T and B cells. Next, the original article of Segatori et al. (2012) conveys preclinical research on racotumomab with or without chemotherapy, and explores the biological role of N-glycolyl gangiosides in a lung cancer mouse model. Two interesting clinical case studies are also part of this book. First, Llanos et al. (2012) report a maintenance treatment with chemotherapy and immunotherapy in a patient with non-small cell lung cancer. Also, Sampor et al. (2012) present results about the immune response to racotumomab in a child with relapsed neuroblastoma. The book closes with a very interesting article by Gómez and Ardigo (2012), analyzing the pharmaceutical perspective of the development of anti-Id Abs in cancer treatment, with a fresh point of view about the relationship between academy and industry. We as editors were very happy to work with such an excellent group of authors, putting together a book with good quality articles that shed light to the use of anti-Id Abs in cancer. Likewise, we hope it constitutes to the reader interesting material for their fields.
